# (*Z*)-{[3-(Hydroxy­meth­yl)-1,3-thia­zolidin-2-yl­idene]amino}formonitrile

**DOI:** 10.1107/S1600536809023095

**Published:** 2009-06-20

**Authors:** Xin-Lin Liu, Yu-Ming Li

**Affiliations:** a Institute of Cardiovascular Disease, Pingjin Hospital, Medical College of Armed Police Force, Tianjin 300162, People’s Republic of China

## Abstract

In the title mol­ecule, C_5_H_7_N_3_OS, all the non-hydrogen atoms except the O atom are almost planar [maximum least squares plane deviation = 0.035 (3) Å for the N atom]. The crystal packing is stabilized by inter­molecular O—H⋯N hydrogen bonds, which link the mol­ecules into inversion dimers.

## Related literature

For a related structure, see: Xie (2008 ▶). For the biological activity of thia­zolidine-containing compounds, see: Iwata *et al.* (1988 ▶). For bond-length data, see: Allen *et al.* (1987 ▶).
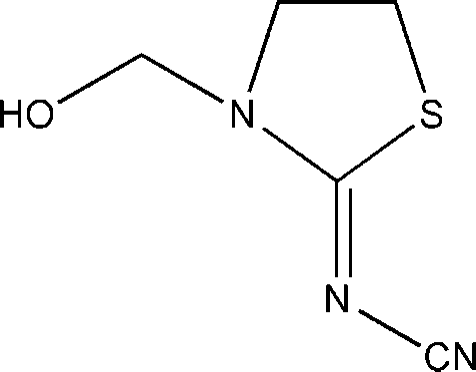


## Experimental

### 

#### Crystal data

C_5_H_7_N_3_OS
*M* *_r_* = 157.20
Triclinic, 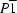

*a* = 5.5321 (11) Å
*b* = 8.1790 (16) Å
*c* = 8.4978 (17) Å
α = 101.56 (3)°
β = 100.39 (3)°
γ = 105.47 (3)°
*V* = 351.75 (16) Å^3^
*Z* = 2
Mo *K*α radiation
μ = 0.39 mm^−1^
*T* = 293 K
0.22 × 0.17 × 0.13 mm

#### Data collection

Rigaku R-AXIS RAPID IP area-detector diffractometer
Absorption correction: multi-scan (*ABSCOR*; Higashi, 1995 ▶) *T*_min_ = 0.919, *T*_max_ = 0.951
2778 measured reflections
1234 independent reflections
1027 reflections with *I* > 2σ(*I*)
*R*_int_ = 0.014

#### Refinement

*R*[*F* ^2^ > 2σ(*F* ^2^)] = 0.032
*wR*(*F* ^2^) = 0.119
*S* = 1.19
1234 reflections
96 parameters
H atoms treated by a mixture of independent and constrained refinement
Δρ_max_ = 0.33 e Å^−3^
Δρ_min_ = −0.25 e Å^−3^

### 

Data collection: *RAPID-AUTO* (Rigaku, 2004 ▶); cell refinement: *RAPID-AUTO*; data reduction: *RAPID-AUTO*; program(s) used to solve structure: *SHELXTL* (Sheldrick, 2008 ▶); program(s) used to refine structure: *SHELXTL*; molecular graphics: *SHELXTL*; software used to prepare material for publication: *SHELXTL*.

## Supplementary Material

Crystal structure: contains datablocks I, global. DOI: 10.1107/S1600536809023095/hg2526sup1.cif

Structure factors: contains datablocks I. DOI: 10.1107/S1600536809023095/hg2526Isup2.hkl

Additional supplementary materials:  crystallographic information; 3D view; checkCIF report

## Figures and Tables

**Table 1:** Hydrogen-bond geometry (Å, °)

*D*—H⋯*A*	*D*—H	H⋯*A*	*D*⋯*A*	*D*—H⋯*A*
O1—H1*A*⋯N3^i^	0.80 (3)	2.04 (3)	2.839 (3)	174 (3)

Symmetry code: (i) 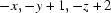.

**Fig. 1.**
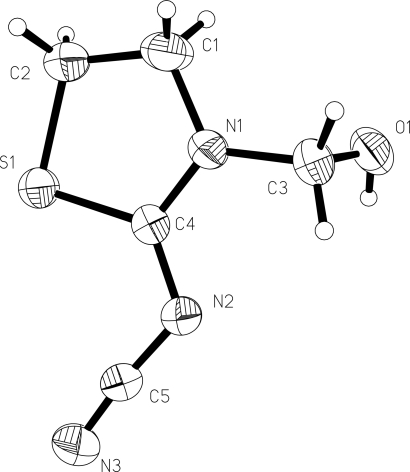
The molecular structure of (I), with atom labels and 40% probability displacement ellipsoids for non-H atoms.

## supplementary crystallographic information

### Comment

Thiazolidine is an important kind of group in organic chemistry. Many compounds containing Thiazolidine groups possess a broad spectrum of biological activities (Iwata *et al.*,  1988). Here, we report the title crystal structure.

In (*Z*)-(3-(hydroxymethyl)thiazolidin-2-ylideneamino)formonitrile (Fig. 1), all bond lengths are normal (Allen *et al.*, 1987) and in a good agreement with those reported previously (Xie, 2008). It is known that the imino tautomers can exist as two geometrical isomers, *syn* (*Z*) and anti (E), but in this crystal, only *Z* isomers have been observed. The atoms of whole molecule except O atom (C1-C5/N1-N3/S1) are almost planar [maximum least squares plane deviation for N1 0.035 (3) Å]. The crystal packing is stabilized by intermolecular O—H···N hydrogen bonds, which link the molecules into dimers.

### Experimental

A mixture of (Z)-(thiazolidin-2-ylideneamino)formonitrile 10 mmol (1.27 g), paraformaldehyde (0.36 g, 12 mmol) and 0.01 g triethylamine were refluxed in absolute EtOH (20 mL) for 3 h. On cooling, the product crystallizes and was filtered and then recrystallized from absolute ethanol. Yield 1.51 g (96%). Single crystals suitable for X-ray measurements were obtained by recrystallization from ethanol at room temperature.

### Refinement

All H atoms were found on difference maps. The hydroxyl H atoms were refined freely, giving an O—H bond distance of 0.80 Å. The remaining H atoms were placed in calculated positions, with C—H = 0.97 Å with *U*_iso_(H) = 1.2 times *U*_eq_(C).

### Crystal data

**Table:**

C_5_H_7_N_3_OS	*Z* = 2
*M**_r_* = 157.20	*F*(000) = 164
Triclinic, *P*1	*D*_x_ = 1.484 Mg m^−^^3^
Hall symbol: -P 1	Mo *K*α radiation, λ = 0.71073 Å
*a* = 5.5321 (11) Å	Cell parameters from 2501 reflections
*b* = 8.1790 (16) Å	θ = 2.3–25.1°
*c* = 8.4978 (17) Å	µ = 0.39 mm^−^^1^
α = 101.56 (3)°	*T* = 293 K
β = 100.39 (3)°	Needle, colorless
γ = 105.47 (3)°	0.22 × 0.17 × 0.13 mm
*V* = 351.75 (16) Å^3^	

### Data collection

**Table:**

Rigaku R-AXIS RAPID IP area-detector diffractometer	1234 independent reflections
Radiation source: Rotating Anode	1027 reflections with *I* > 2σ(*I*)
graphite	*R*_int_ = 0.014
ω oscillation scans	θ_max_ = 25.0°, θ_min_ = 3.2°
Absorption correction: multi-scan (*ABSCOR*; Higashi, 1995)	*h* = −6→6
*T*_min_ = 0.919, *T*_max_ = 0.951	*k* = −9→9
2778 measured reflections	*l* = −10→10

### Refinement

**Table:**

Refinement on *F*^2^	Secondary atom site location: difference Fourier map
Least-squares matrix: full	Hydrogen site location: inferred from neighbouring sites
*R*[*F*^2^ > 2σ(*F*^2^)] = 0.032	H atoms treated by a mixture of independent and constrained refinement
*wR*(*F*^2^) = 0.119	*w* = 1/[σ^2^(*F*_o_^2^) + (0.0703*P*)^2^ + 0.06*P*] where *P* = (*F*_o_^2^ + 2*F*_c_^2^)/3
*S* = 1.19	(Δ/σ)_max_ < 0.001
1234 reflections	Δρ_max_ = 0.33 e Å^−^^3^
96 parameters	Δρ_min_ = −0.25 e Å^−^^3^
0 restraints	Extinction correction: *SHELXTL* (Sheldrick, 2008), Fc^*^=kFc[1+0.001xFc^2^λ^3^/sin(2θ)]^-1/4^
Primary atom site location: structure-invariant direct methods	Extinction coefficient: 0.052 (16)

### Special details

**Table:**

Geometry. All esds (except the esd in the dihedral angle between two l.s. planes) are estimated using the full covariance matrix. The cell esds are taken into account individually in the estimation of esds in distances, angles and torsion angles; correlations between esds in cell parameters are only used when they are defined by crystal symmetry. An approximate (isotropic) treatment of cell esds is used for estimating esds involving l.s. planes.
Refinement. Refinement of F^2^ against ALL reflections. The weighted R-factor wR and goodness of fit S are based on F^2^, conventional R-factors R are based on F, with F set to zero for negative F^2^. The threshold expression of F^2^ > 2sigma(F^2^) is used only for calculating R-factors(gt) etc. and is not relevant to the choice of reflections for refinement. R-factors based on F^2^ are statistically about twice as large as those based on F, and R- factors based on ALL data will be even larger.

### Fractional atomic coordinates and isotropic or equivalent isotropic displacement parameters (Å^2^)

**Table:**

	*x*	*y*	*z*	*U*_iso_*/*U*_eq_	
S1	0.15141 (12)	0.91511 (8)	0.84635 (8)	0.0509 (3)	
O1	0.3210 (4)	0.3944 (2)	0.6397 (3)	0.0640 (6)	
N1	0.3875 (3)	0.6968 (2)	0.7503 (2)	0.0450 (5)	
N2	0.2714 (4)	0.6719 (2)	0.9942 (2)	0.0490 (5)	
N3	0.0736 (5)	0.7702 (3)	1.2185 (3)	0.0640 (6)	
C1	0.3729 (7)	0.7839 (5)	0.6182 (4)	0.0724 (9)	
H1B	0.2783	0.6980	0.5138	0.087*	
H1C	0.5460	0.8400	0.6090	0.087*	
C2	0.2400 (6)	0.9180 (4)	0.6529 (3)	0.0592 (7)	
H2B	0.0866	0.8916	0.5642	0.071*	
H2C	0.3545	1.0335	0.6600	0.071*	
C3	0.4959 (5)	0.5525 (3)	0.7414 (3)	0.0529 (6)	
H3A	0.6495	0.5809	0.6993	0.063*	
H3B	0.5477	0.5389	0.8521	0.063*	
C4	0.2782 (4)	0.7467 (3)	0.8711 (3)	0.0395 (5)	
C5	0.1620 (5)	0.7289 (3)	1.1096 (3)	0.0482 (6)	
H1A	0.216 (6)	0.353 (4)	0.686 (4)	0.080 (10)*	

### Atomic displacement parameters (Å^2^)

**Table:**

	*U*^11^	*U*^22^	*U*^33^	*U*^12^	*U*^13^	*U*^23^
S1	0.0639 (4)	0.0564 (4)	0.0506 (4)	0.0356 (3)	0.0251 (3)	0.0217 (3)
O1	0.0818 (13)	0.0500 (10)	0.0697 (13)	0.0227 (9)	0.0443 (11)	0.0109 (9)
N1	0.0543 (11)	0.0508 (10)	0.0416 (11)	0.0272 (9)	0.0206 (9)	0.0158 (8)
N2	0.0661 (12)	0.0492 (11)	0.0457 (12)	0.0278 (9)	0.0249 (9)	0.0202 (9)
N3	0.0854 (16)	0.0641 (13)	0.0531 (14)	0.0263 (12)	0.0342 (12)	0.0195 (11)
C1	0.101 (2)	0.102 (2)	0.0584 (17)	0.0688 (19)	0.0462 (16)	0.0452 (16)
C2	0.0827 (17)	0.0620 (15)	0.0481 (15)	0.0335 (14)	0.0262 (13)	0.0240 (12)
C3	0.0601 (14)	0.0561 (14)	0.0562 (15)	0.0318 (12)	0.0255 (12)	0.0166 (12)
C4	0.0416 (11)	0.0386 (10)	0.0389 (12)	0.0140 (9)	0.0111 (9)	0.0087 (9)
C5	0.0622 (14)	0.0462 (12)	0.0421 (14)	0.0191 (11)	0.0171 (11)	0.0179 (10)

### Geometric parameters (Å, °)

**Table:**

S1—C4	1.735 (2)	N3—C5	1.156 (3)
S1—C2	1.801 (3)	C1—C2	1.486 (4)
O1—C3	1.392 (3)	C1—H1B	0.9700
O1—H1A	0.80 (3)	C1—H1C	0.9700
N1—C4	1.331 (3)	C2—H2B	0.9700
N1—C1	1.447 (3)	C2—H2C	0.9700
N1—C3	1.455 (3)	C3—H3A	0.9700
N2—C5	1.314 (3)	C3—H3B	0.9700
N2—C4	1.315 (3)		
			
C4—S1—C2	92.28 (11)	S1—C2—H2B	110.0
C3—O1—H1A	111 (2)	C1—C2—H2C	110.0
C4—N1—C1	116.2 (2)	S1—C2—H2C	110.0
C4—N1—C3	122.77 (19)	H2B—C2—H2C	108.4
C1—N1—C3	120.8 (2)	O1—C3—N1	112.3 (2)
C5—N2—C4	118.1 (2)	O1—C3—H3A	109.1
N1—C1—C2	110.0 (2)	N1—C3—H3A	109.1
N1—C1—H1B	109.7	O1—C3—H3B	109.1
C2—C1—H1B	109.7	N1—C3—H3B	109.1
N1—C1—H1C	109.7	H3A—C3—H3B	107.9
C2—C1—H1C	109.7	N2—C4—N1	121.5 (2)
H1B—C1—H1C	108.2	N2—C4—S1	125.35 (17)
C1—C2—S1	108.30 (18)	N1—C4—S1	113.20 (17)
C1—C2—H2B	110.0	N3—C5—N2	174.2 (3)
			
C4—N1—C1—C2	1.7 (4)	C1—N1—C4—N2	177.4 (2)
C3—N1—C1—C2	176.5 (2)	C3—N1—C4—N2	2.8 (3)
N1—C1—C2—S1	−0.2 (3)	C1—N1—C4—S1	−2.5 (3)
C4—S1—C2—C1	−0.9 (2)	C3—N1—C4—S1	−177.14 (17)
C4—N1—C3—O1	95.2 (3)	C2—S1—C4—N2	−178.0 (2)
C1—N1—C3—O1	−79.2 (3)	C2—S1—C4—N1	1.95 (18)
C5—N2—C4—N1	179.5 (2)	C4—N2—C5—N3	−171 (3)
C5—N2—C4—S1	−0.6 (3)		

### Hydrogen-bond geometry (Å, °)

**Table:**

*D*—H···*A*	*D*—H	H···*A*	*D*···*A*	*D*—H···*A*
O1—H1A···N3^i^	0.80 (3)	2.04 (3)	2.839 (3)	174 (3)

Symmetry codes: (i) −*x*, −*y*+1, −*z*+2.
